# Evaluating a Model of Added Sugar Intake Based on Amino Acid Carbon Isotope Ratios in a Controlled Feeding Study of U.S. Adults

**DOI:** 10.3390/nu14204308

**Published:** 2022-10-14

**Authors:** Jessica J. Johnson, Virág Sági-Kiss, Susana A. Palma-Duran, John Commins, Matthew Chaloux, Brian Barrett, Douglas Midthune, Victor Kipnis, Laurence S. Freedman, Natasha Tasevska, Diane M. O’Brien

**Affiliations:** 1 Institute of Arctic Biology, Department of Biology and Wildlife, University of Alaska Fairbanks, Fairbanks, AK 99775, USA; 2 College of Health Solutions, Arizona State University, Phoenix, AZ 85004, USA; 3 Information Management Services, Inc., Rockville, MD 20850, USA; 4 Division of Cancer Prevention, National Cancer Institute, Bethesda, MD 20892, USA; 5 Gertner Institute for Epidemiology and Health Policy Research, Sheba Medical Center, Tel Hashomer, Ramat Gan 52621, Israel

**Keywords:** alanine, dietary biomarkers, carbon stable isotope ratios, animal protein ratio, controlled feeding study

## Abstract

Previous studies suggest that amino acid carbon stable isotope ratios (CIR_AA_s) may serve as biomarkers of added sugar (AS) intake, but this has not been tested in a demographically diverse population. We conducted a 15-day feeding study of U.S. adults, recruited across sex, age, and BMI groups. Participants consumed personalized diets that resembled habitual intake, assessed using two consecutive 7-day food records. We measured serum (*n* = 99) CIR_AA_s collected at the end of the feeding period and determined correlations with diet. We used forward selection to model AS intake using participant characteristics and 15 CIR_AA_s. This model was internally validated using bootstrap optimism correction. Median (25th, 75th percentile) AS intake was 65.2 g/day (44.7, 81.4) and 9.5% (7.2%, 12.4%) of energy. The CIR of alanine had the highest, although modest, correlation with AS intake (*r* = 0.32, *p* = 0.001). Serum CIR_AA_s were more highly correlated with animal food intakes, especially the ratio of animal to total protein. The AS model included sex, body weight and 6 CIR_AA_s. This model had modest explanatory power (multiple *R^2^* = 0.38), and the optimism-corrected *R^2^* was lower (*R^2^* = 0.15). Further investigations in populations with wider ranges of AS intake are warranted.

## 1. Introduction

There is a crucial need in the field of nutritional epidemiology for biomarkers of added sugar (AS) intake, which has been linked with obesity and chronic disease [1,2,3,4], but remains challenging to measure [5,6]. Objective dietary biomarkers that have a stable relationship with dietary intake may be used to calibrate self-reported intakes and assess inherent systematic measurement errors, and thus, improve diet–disease risk models [7,8]. For example, the twenty-four-hour urinary sucrose and fructose (24uSF) biomarker has been developed to calibrate total sugars intake in various populations [9,10,11]; however, 24uSF does not distinguish intrinsic from added sugars. Stable carbon isotope ratios (CIRs), which are naturally elevated in corn and sugarcane [12,13], may differentiate these sources of sugar from others, especially when measured in compounds involved in sugar metabolism.

The nonessential amino acid (NEAA) alanine (Ala) is central to glucose metabolism [14,15], and the CIR_Ala_ has been correlated with the intake of AS generally, and sugar-sweetened beverages (SSBs) specifically, in several studies [16,17,18]. In one cross-sectional study, red blood cell (RBC) CIR_Ala_ was highly correlated with AS and SSB intakes, and not with the intake of meat [16], which can be another source of elevated CIRs in the U.S. diet due to corn-based animal feeds [12,19,20]. In a highly controlled, inpatient feeding study of adult men, the CIR_Ala_, among other CIR_NEAA_s, was elevated in response to SSB, and again, not meat intake. In a report on the Nutrition and Physical Activity Assessment Study Feeding Study (NPAAS-FS) of the Women’s Health Initiative, the authors noted that the CIR_Ala_ was positively correlated with both AS and animal protein intakes but that other CIR_AA_s were positively correlated with animal protein and either uncorrelated or negatively correlated with AS. They then developed a model of AS intake that included multiple serum CIR_AA_s (CIR_Ala_, CIR_Gly_, CIR_Ile_) and participant characteristics [17]. The resulting AS model had a higher *R^2^* than the CIR_Ala_ alone and was not associated with animal protein. This suggests that multiple CIR_AA_s should be considered when explaining or predicting AS intake, as this inclusion may help control for the error in the CIR_Ala_ relationship that is due to animal protein intake or due to CIR_AA_ measurement error.

In this study, we aimed to model AS intake in a controlled feeding study of U.S. adults, recruited across sex, age, and BMI groups [21] using CIR_AA_s measured in participant serum samples. The present study population is more demographically diverse than those from the previous studies described above, the goal of which was to make the results more generalizable. However, after the analysis of whole serum isotope ratios, we found that this population had a particularly high protein intake, and that serum carbon and nitrogen isotope ratios were highly correlated with animal protein intake [22]. Following the NPAAS-FS, we anticipated that the CIR_Ala_ would be more highly correlated with AS intake than whole serum isotope ratios and that other CIR_AA_s could help account for animal protein intake [17]. We proceeded with a modelling approach similar to that used in the NPAAS-FS cohort by selecting multiple CIR_AA_s and participant characteristics to explain AS intake. We then explored internal validation of this modelling approach using bootstrapping and compared it to alternative model selection approaches.

## 2. Materials and Methods

### 2.1. Study Participants

Participants were recruited to a controlled feeding study, designed to test biomarkers of total and added sugars intake. The primary outcomes reported in the present analysis are the responses of CIR_AA_s to AS intake. Adult (18–70 years) men and women (*n* = 113) were recruited from the Phoenix Metropolitan Area between March 2016 and May 2019, as described in detail elsewhere [21]. Study eligibility was limited to participants with a BMI <35 kg/m^2^, and exclusion criteria included the following: smoking, kidney disease, disorders that affect energy metabolism (e.g., thyroid disease, liver disease, and cancer), pregnancy or breastfeeding, any dietary restriction due to a medical condition, weight loss in the previous 4 months, and fasting blood glucose ≥ 100 mg/dl or HbA1c ≥ 5.7% upon screening. Participant recruitment was stratified across sex, age, and BMIs to obtain a heterogeneous sample population. The study was approved by the Institutional Review Board of Arizona State University (ID: STUDY00002695), which approved that it did not meet the NIH criteria for a clinical trial. All participants signed written informed consent forms.

### 2.2. Study Design

We conducted a 15-day controlled feeding study with personalized diets based on each participant’s habitual diet [21,22]. Participants were recruited using hard copy and web-based postings, as well as e-mail (e.g., Arizona State University) lists. We first screened respondents using an online questionnaire, and at a follow-up screening visit, we measured body weight and height, and collected a fasting blood sample to measure blood glucose and HbA1c. We accepted eligible participants into the study according to a stratification plan designed to ensure variation in sex (male and female), age (18–34, 35–44, 45–54, and 55–70 years), and BMI (<25, 25–29.9, and 30–34.9) across the study population.

Self-reported demographics, lifestyle, and medical history were collected with a baseline questionnaire, and habitual dietary intakes were determined using 7-day food records. Participants received training on keeping a 7-day food record and were then instructed to record all foods and beverages consumed over a 2-week period while maintaining their usual diets. Two weekly interviews were conducted with participants to capture details about specific foods, recipes, and cooking methods. Food records were used to design 15-day diets for each participant; all meals were prepared in the metabolic kitchen at the Arizona State University study center. Meals were made with foods recorded in the Nutrition Data System for Research (NDSR) database (Nutrition Coordinating Center, Minneapolis, MN). We substituted foods with comparable sugar content for brand name foods that were not available in the NDSR. If no comparable foods were available, we worked with the NDSR to add new food records based on the food company-provided nutrient and ingredient content of the missing food.

Participants began the 15-day feeding period one week after completing their food records. They visited the metabolic kitchen daily, between Monday and Friday, to consume either breakfast or lunch and to pick up their additional meals until the next visit. Weekend meals were collected on Fridays. Participants were given ~1.25 times the amount of food reported in their food records and were allowed to consume as much as they wanted. They were asked to return any unconsumed foods in the original packaging; these were weighed and subtracted from the amount provided to obtain the amount consumed. Participants were instructed not to consume anything outside of the provided foods, except for water, coffee, tea, and alcohol (but not liqueurs). Any sweeteners or creamers consumed with coffee or tea were provided by the study. Each participant kept a daily meal log, where they recorded any deviations from the feeding protocol and details on the amounts and types of tea, coffee, and alcohol consumed. Consumed foods were entered into the NDSR to calculate daily energy and nutrient intakes. The NDSR had analytical values for AS for 78% of core foods, and the rest were imputed values [23].

Participant body weight and height were measured daily following a standardized protocol. The fasting blood samples used to measure serum CIR_AA_s were collected from participants on the morning after the last day of the 15-day feeding study. Fasting blood was also collected at the beginning of the feeding period (baseline) and 5 weeks after the completion of the feeding period. The decision to analyze only the end-of-feeding sample was based on the very high correlations of whole serum CIRs between the time points [22]. Blood for serum isolation was collected in red top, gel-barrier tubes. Serum samples were frozen at −80 °C and shipped to the University of Alaska Fairbanks for stable isotope analyses. Samples were anonymized for analytical measurements.

### 2.3. Biomarker Measures: Amino Acid Carbon Isotope Ratios

Serum CIR_AA_s were measured using gas chromatography-combustion-isotope ratio mass spectrometry (GC-C-IRMS) and are reported as δ^13^C values with units of per mil (‰), as follows: δ^13^C = (^13^C/^12^C_sample_/^13^C/^12^C_reference_ − 1) × 1000‰, where the reference is Vienna Pee Dee Belemnite (^13^C/^12^C = 0.0112372), an established international reference material for δ^13^C measurements. We retain CIR_AA_ as the variable name when referring to amino acid δ^13^C values. We prepared and analyzed serum samples (*n* = 99) in randomized batches that were balanced by sex, age, and BMI and that were checked to prevent biasing of AS intake (i.e., no batch was made up of exclusively low or high AS consumers). The analyst was blinded to these variables during sample analysis.

Amino acids were hydrolyzed and derivatized prior to measurement of CIRs via GC-C-IRMS, as described in detail elsewhere [18]. Serum samples were hydrolyzed using HCl (6 mol/L) and lipid-extracted using n-hexane and dichloromethane (6:5 vol:vol). Dried AA hydrolysates were derivatized to N-acetyl methyl esters in two steps: methylation with acidified methanol and acetylation using a mixture of acetic anhydride, triethylamine, and acetone (1:2:5 vol:vol:vol). Derivatized AAs were purified with a phosphate buffer wash (1 mol/L potassium phosphate + 1 mol/L sodium phosphate, pH 7) and extracted using chloroform. Purified AA derivatives were dried, dissolved in ethyl acetate, and stored at −18 °C until GC-C-IRMS analysis. Each batch included an external standard, containing a mix of commercial AAs (Table S1) with known non-derivatized CIRs, and a laboratory serum check sample. We added three internal standards to all external standards and samples: one requiring derivatization (norleucine) and two that are volatile (nonadecane and caffeine). Internal standards were used to monitor instrument performance but were not used to adjust measured CIR_AA_s.

GC-C-IRMS analyses were performed at the Alaska Stable Isotope Facility at the University of Alaska Fairbanks using a GC IsoLink II System (Thermo Fisher Scientific, Waltham, MA, USA). Derivatized AAs were injected onto a VF-35ms column (Agilent, Santa Clara, CA, USA) in a TRACE 1310 GC (Thermo Fisher Scientific, Waltham, MA, USA) for peak separation, and the GC effluent was routed through the Isolink II interface for combustion of each individual AA into CO_2_ gas and introduction into a Delta V Plus isotope ratio mass spectrometer (Thermo Fisher Scientific, Waltham, MA, USA) for determination of CIRs. Each sample was analyzed in triplicate injections. An injection of the external standard was made between triplicates (*n* = 13–16 per analytical sequence).

CIR_AA_s are reported in delta notation (δ^13^C values) with units of per mil (‰) as described above, using calibrated CO_2_ gas as the proximal reference material for Vienna Pee Dee Belemnite. The ^13^C/^12^C ratio is calculated by peak integration in the program Isodat (version 3.0, Thermo Scientific, Waltham, MA, USA). Correct peak identification and integration (width and background assignment) and adequate separation between peaks was visually confirmed, and manually adjusted as necessary. We obtained reliable chromatography for 15 AAs: alanine (Ala), aspartic acid/asparagine (Asx), glutamic acid/glutamine (Glx), glycine (Gly), histidine (His), isoleucine (Ile), leucine (Leu), lysine (Lys), methionine (Met), phenylalanine (Phe), proline (Pro), serine (Ser), threonine (Thr), tyrosine (Tyr), and valine (Val). Asparagine and glutamine are deamidated to aspartic acid and glutamic acid, respectively, during acid hydrolysis; thus, their respective CIR values are combined measurements.

The derivatization process used here adds carbon atoms to AAs and causes potential kinetic isotope effects, both of which influence the CIRs of derivatized AAs. These influences are accounted for by adjusting the measured CIR of derivatized AAs in samples using the measured CIR of derivatized AAs in the external standard. The measured CIR_AA_s of derivatized AAs (termed CIR_AA,d_) were adjusted using the known CIR_AA_ in the external standard, as follows:

(1) $$CIR_{AA\left(smp\right)}=\frac{\left(CIR{\;}_{AA,d\left(smp\right)}-\;CIR_{AA,d\left(std\right)}\right)}{p}+CIR{\;}_{AA\left(std\right)}$$

where CIR_AA,d(smp)_ and CIR_AA,d(std)_ are the measured CIR of the derivatized AA in the sample and external standard, respectively; CIR_AA(std)_ is the known value of the CIR_AA_ in the external standard; p is the proportion of carbon in the derivatized AA from the un-derivatized AA; and CIR_AA(smp)_ is the corrected sample CIR_AA_.

The propagated analytical error (SEM) of CIR_AA_ measurements was estimated for triplicate injections of the check sample, as described elsewhere [24] and in the Table S2. This within-batch error ranged from 0.05‰ for the CIR_Phe_ to 0.33‰ for the CIR_His_. We used measurements of the check sample across batches to evaluate reproducibility. This between-batch reproducibility ranged from 0.18‰ for the CIR_Phe_ to 1.33‰ for the CIR_Gly_ with an average of 0.69‰. Analytical error and reproducibility of the check sample for all CIR_AA_s are reported in Table S2.

### 2.4. Statistical Analyses

We calculated the Pearson correlation coefficients between CIR_AA_s and 15-day mean dietary intake variables, including total carbohydrate (g/day), AS (g/day), SSBs (servings/day), total protein (g/day), animal protein (g/day), various categories of animal-derived foods (g/day), and corn products (g/day). The derivation of the corn products and other food group variables is detailed elsewhere [17]. The linear relationships between variables were visually assessed, and correlations were calculated on transformed variables where linearity was improved. These and the following analyses were performed in R version 4.0.1.

We selected a multiple linear regression model to explain AS intakes (*n* = 99) using a forward selection approach on participant characteristics and CIR_AA_ biomarker variables. Final variable stepwise addition was based on the condition that the change in the Akaike information criterion (AIC) should be greater than 2. We used the stepAIC function from the MASS package [25] to select participant characteristics (age, sex, and body weight) first. Selected participant characteristics then formed the base model for forward selection of CIR_AA_ variables. We ran a bootstrap procedure to internally validate the model and calculate Harrell’s optimism-corrected *R^2^* [26]. We used the boot.stepAIC function from the boot.stepAIC package to perform forward stepwise selection of participant and CIR_AA_ variables on 2,000 bootstrap samples of the original data set. From these, model stability was assessed based on the frequency with which CIR_AA_ variables were selected.

We also explored whether two other model selection procedures, least absolute shrinkage and selection operator (LASSO) regression [27] and random forest regression [28], might perform better than forward selection. We used mean square prediction error (MSPE) to compare the three approaches. The details of these analyses are in Appendix A.

## 3. Results

### 3.1. Study Participants and Diets

There were 113 participants who enrolled in the study, and 100 completed the entire feeding protocol (Figure S1). However, 1 completed participant’s serum sample was not included in the current study, because it was separated in error during storage, then analyzed under different conditions from the other 99 samples, and had outlying CIR_AA_ values. Of the participants whose CIR_AA_s were measured (*n* = 99), 55% of them were female (Table 1). The majority of participants (78%) identified as non-Hispanic White, and the remaining participants identified as Hispanic/Latino (*n* = 12), African American (*n* = 3), Pacific Islander (*n* = 1), Native American (*n* = 1), Asian (*n* = 4), or did not self-identify (*n* = 1). The median age was 38 years, and the distribution by age category was: 18–34 years (*n* = 40), 35–44 years (*n* = 19), 45–54 years (*n* = 19), and 55–70 years (*n* = 21). There was an even distribution of older (≥45 years) and younger (<45 years) females, but twice as many younger compared with older males. The median BMI was 26.9, and the distribution was balanced among the recruited BMI categories: <25 (*n* = 33), 25–29.9 (overweight, *n* = 36), and 30–34.9 (obese, *n* = 30), and across sexes.

The AS intake of participants ranged from 9.3 g/day to 136.8 g/day (median 60.2 g/day) for females and 10.7 g/day to 210.8 g/day (median 66.4 g/day) for males. AS intake as a percent of daily energy ranged from 1.7% to 21.9% (median 9.8%) for females and 1.5% to 19.3% (median 8.9%) for males. The ratio of added to total sugar intake, the added sugar ratio (ASR), was also similar for females (median ± IQR, 0.58 ± 0.21) and males (median ± IQR, 0.60 ± 0.20). Intake of sugar-sweetened beverages (SSBs) was low: 15% of participants had no intake, and the median intake was 0.42 ± 0.86 servings/day.

Median (±IQR) animal protein intake was 58.7 ± 25.5 g/day for females and 84.5 ± 32.8 g/day for males. The ratio of animal to total protein intake, the animal protein ratio (APR), was similar for females (0.64 ± 0.12) and males (0.67 ± 0.08). There was a very low correlation between AS intake and animal protein intake (Pearson *r* = 0.05, *p* = 0.61). However, there were modest negative correlations between non-added sugar (total—added sugar) and the APR (Pearson *r* = −0.38, *p* < 0.001) and between non-sugar carbohydrate intake (g/day) and the APR (Pearson *r* = −0.30, *p* = 0.002).

There were moderate to high positive correlations within participant CIRAAs, which are shown in Table S3.

### 3.2. Biomarker Correlations with Diet

Correlations between serum CIR_AA_s and AS intake were overall low (Table 2 and Table 3), the highest correlation being with the CIR_Ala_ (Pearson *r* = 0.32, *p* = 0.001). There were modest correlations of CIR_NEAA_s with non-added sugar intake (negative) and the ASR (Table 2). There were also modest negative correlations among the CIRs of EAAs with total minus added sugar intake, and the CIR_Lys_ also had a modest positive correlation with the ASR (Table 3).

The highest correlations between serum CIR_AA_s and diet were between CIR_EAA_s and the animal protein ratio (APR), the highest of these being with the CIR_Leu_ (Pearson *r* = 0.84, *p* < 0.0001) and the CIR_Phe_ (Pearson *r* = 0.85, *p* < 0.0001) (Table 3). Multiple CIR_EAA_s were correlated with both animal (positive) and plant (negative) protein intakes. CIR_NEAA_s were also more correlated with the APR, plant protein intake, and animal protein intake (except for the CIR_Ala_) than with any of the sugar-related intakes. Red meat intake had the highest correlations of any food group with CIR_AA_s, including most CIR_EAA_s and CIR_NEAA_s. Poultry intake also had modest correlations with several CIR_EAA_s and CIR_NEAA_s.

### 3.3. Model Selection for Added Sugar Intake

The final model for AS intake included body weight, sex, and 6 CIR_AA_s (4 CIR_NEAA_s and 2 CIR_EAA_s) (Table 4), and was modestly explanatory (*R^2^* = 0.38, adjusted *R^2^* = 0.32). The CIR_AA_ with the largest β coefficient was the CIR_Ala_, and it was positive. The bootstrap optimism-corrected *R^2^* for the model was low (corrected *R^2^* = 0.15). Among bootstrap forward selected models, the CIR_Ala_ was the most frequently selected CIR_AA_ covariate (98% of models) (Table S4). The other CIR_AA_ covariates had a range of selection frequencies across bootstrap models, with the CIR_Ser_ having a selection frequency of only 46%. The average MSPEs for models selected using forward selection, LASSO, and random forest were similar, and MSPEs for each run were variable across methods (Table A1). Due to the correlation of the CIR_Ala_ with the ASR, we also explored forward selection of a model explaining ASR, but it was not superior to the AS model (results not shown).

## 4. Discussion

In this controlled feeding study of 99 men and women of varying age and BMI, we explored how well serum CIR_AA_s could explain AS intake. After adjustment for multiple testing, only one CIR_AA_, the CIR of Ala, was statistically significantly correlated with AS intake (*r* = 0.32), while most CIR_AA_s were correlated with animal protein intake and the animal protein ratio (APR). The model chosen to describe AS intake using the forward selection approach included multiple CIR_AA_s, along with participant sex and body weight. This model was modestly explanatory of the data used for its development but was less so in internal bootstrap validation.

The CIR of a particular serum AA results from the CIR of that same AA or any of its precursors in the diet. In the case of EAAs, their CIRs are a proportionate mix of CIR_EAA_s deriving from dietary animal and plant protein, with higher and lower CIRs, respectively. This is demonstrated in the present study in both the negative correlations of CIR_EAA_s with plant protein and the positive correlations with the APR. In the case of NEAAs, which can be synthesized from non-AA precursors, their CIRs will reflect a more complicated mix of dietary protein and non-protein sources. In this study, although CIR_NEAA_s were highly influenced by animal and plant protein intakes, the CIR_Ala_ was also influenced by AS intake and the ASR, as expected from the metabolic link between glucose and Ala [14,15]. Furthermore, the correlations of CIR_NEAA_s, including the CIR_Ala_, with the ASR were all higher than those with AS alone, demonstrating again that CIR_NEAA_s result from both higher-CIR added, and lower-CIR non-added, sugars. However, we caution that there was also a negative correlation of total minus added sugar (non-added sugar) with the APR (r = −0.38), which may partially explain the correlation of CIR_NEAA_s with non-added sugar.

The results of this study are similar to those from a similar 2-week controlled feeding study based on usual intake in postmenopausal women, the NPAAS-FS [17,29]. In both studies, the CIR_Ala_ was the only CIR_AA_ to be positively correlated with AS intake (Spearman *ρ* = 0.32 in the NPAAS-FS) [17], demonstrating improved sensitivity to AS over the whole serum CIR (*r* = 0.02 in the NPAAS-FS and *r* = 0.05 in the current study population) [22,29]. In both studies, the CIR_Ala_ was also affected by protein intake: the correlation with APR was not reported in the NPAAS-FS, but there was a significant correlation with animal protein intake (Spearman *ρ* = 0.23) [17], similar to what we found in this study (*r* = 0.25). The model for AS intake derived from the NPAAS-FS data contained multiple CIR_AA_s and participant characteristics, as in the current study, and was modestly explanatory (*R^2^* = 0.37) [17]. The ratio of median AS intake to AP intake was very similar in the two studies: 48 (35, 63) g/day of AS and 49 (41, 59) g/day of AP in the NPAAS-FS and 65 (45, 81) g/day of AS and 67 (55, 88) g/day of AP in the current study. The intake of SSBs, substantial sources of AS in other populations, was similarly low in both studies. Sugarcane, corn, and corn-fed livestock are the primary sources of elevated CIRs in the diet [12,19,30], and it is thus possible that the similar trends in AS and AP intakes resulted in the similar CIR_Ala_ and CIR_AA_ responses.

While modest in the present study and in the NPAAS-FS, the association of the CIR_Ala_ with AS intake has been higher in previous studies. In a highly controlled, inpatient feeding study, where both SSB and meat intakes were varied at the same time, CIRs were elevated in Ala (β = 2.81, SE = 0.38) and multiple other N_EAA_s in response to SSB but not meat intake [18]. There were also high and moderately high correlations of the CIR_Ala_ with both SSB (*r* = 0.70) and AS (*r* = 0.59) intakes in a cross-sectional study of men and women from two Yup’ik villages in Alaska [16]. In both of these previous studies, AS and SSB intakes were higher than in the current study. It is possible that CIR_NEAA_s, and the CIR_Ala_ in particular, correlate more strongly to SSBs than total dietary AS. The AS in SSBs derives predominantly from sugarcane and corn, while AS in other foods may come from additional sources (e.g., honey, maple, and beet) that do not have elevated CIRs [12]. It is also possible that AS in SSBs versus solid foods differentially affect whole tissue CIRs and CIR_AA_s, as there are also questions about differential metabolic impacts of AS in liquid and solid foods [31]. The drivers of the apparent discrepancies across studies with varying AS intake patterns and CIR_AA_ responses warrant further consideration.

In conjunction with lower AS intake, the APR may have constrained the ability of our CIR_AA_-based model to describe AS intake in this population. The estimated proportion of plasma Ala derived from plasma glucose is ~40%, and the remainder derives from Ala and other AAs in dietary and tissue protein [14,15]. Thus, the CIR_Ala_ was likely influenced by the high proportion of AP in this study [median APR = 0.66 (0.59, 0.70)]. As expected, the correlations of APR and most CIR_EAA_s were high. In fact, these results indicate that single or multiple CIR_EAA_ biomarkers may perform as well as the serum CIR biomarker from the same study in predicting APR [22]. The inclusion of CIR_EAA_s in the model likely controlled for some of the association between the CIR_Ala_ and APR. However, given how well CIR_EAA_s describe APR, high APR intakes cannot be the only reason for the modest explanatory power of the AS model.

Another possible reason for the modest explanatory power in the AS model is measurement error in the CIR_AA_ values. The reproducibility of the CIR measurement varied across AAs, as demonstrated by the batch-to-batch variability of a quality assurance serum check sample. Greater measurement error may attenuate correlations of CIR_AA_s with diet [32]. Indeed, we found that the CIR_EAA_s with the highest between-batch variability also had the lowest correlations with APR. The measurement error of the CIR_Ala_ was lower than that of the majority of the CIR_AA_s measured; however, it was still twice as high as the error of the most precisely measured CIR_EAA_. Correlated measurement errors among CIR_AA_s may also explain the selection of CIR_AA_ covariates that did not have significant associations with AS intake.

There are recognized limitations to a model chosen by forward selection which may have been compounded by the nature of our data [33]; thus, we also explored alternative approaches to modelling AS intake. There were multiple high correlations among CIR_AA_s, reflecting the fact that they are measured at the same time and are therefore not independent. The variability of covariate selection in the bootstrap forward selection demonstrates model instability (Table S4). However, neither LASSO nor the random forest approach decreased the mean square prediction error of our model. Random forest is a machine learning technique that does not make assumptions about the form of the model and can therefore handle interactions and nonlinear relationships among covariates. In addition, it is an ensemble technique which develops a more robust and stable final prediction as an average of multiple tree models using bootstrap samples from the original data. The random forest approach should have reduced the MSPE in the case of interactions and nonlinearities, although our sample size did limit its efficacy. LASSO is more robust to multiple collinearities than other regression approaches [27]; however, we did not see an improvement in MSPE with this method, either. This suggests that we were not limited by the forward selection approach to developing an AS model, but by the underlying relationship between AS intake and the measured CIR_AA_s, the low intake of AS in the study cohort, and also by the limited sample size.

Despite the limitations described above, the CIR_Ala_, alone or in combination with other CIR_AA_s, shows promise as a biomarker for AS or SSB intake in certain populations and warrants further study [34]. The only other proposed biomarker for AS intake is whole-tissue CIRs, which have been shown to correlate more with meat or animal protein intakes in various studies across populations [20,22,29,35]. Blood CIR has been indicated as a candidate biomarker of AS and SSB intake in several Virginia-based studies [36,37,38]. However, meat and animal protein intake were not considered in at least two of these studies [36,37], and in a third, the AS intake was almost twice as large as animal protein intake [38]. Furthermore, the prediction model for AS intake developed from blood CIR demonstrated poor performance in external validation; specifically, high intakes were underestimated [39]. There has been some metabolomic exploration of SSB intake biomarkers [40], but this approach has the limitation of identifying compounds that may not be present in all SSBs (low sensitivity) or may be present in other foods (low specificity) [34]. Metabolomic biomarkers are also often limited to indicating short-term intake, whereas plasma and RBC CIR_AA_s integrate diet over several weeks or months [18]. Recently, a positive association between AS intake and the CIR_Ala_ of RBCs was found in a majority Māori population in New Zealand, suggesting the robustness of the CIR_Ala_ across populations with sugarcane and corn based AS [41]. All this supports the continued study of CIR_AA_ biomarkers of AS and SSB intake, especially in populations with wide-ranging intakes.

The main strengths of this study were the controlled feeding design and the demographically diverse study population. The feeding protocol approximated participants’ usual diets and was controlled for a 15-day period, which increased the reliability of actual intake. It also simulated intake of a free-living population, allowing us to assess the robustness of the dietary biomarkers [42]. Recruitment was stratified across sexes, ages, and BMIs, making this study population more representative of the general U.S. population than prior studies where CIR_AA_s have been measured. The main limitation of this study was that the recruited population did not reflect a wide range of AS or SSB intake. There were also analytical limitations to measuring CIR_AA_s: we were able to measure certain CIR_AA_s with greater precision than others. Finally, our sample size limited how well we could determine out-of-sample MSPE and compare different model selection approaches.

## 5. Conclusions

In conclusion, we were able to explain AS intake to a modest extent in a controlled feeding study of U.S. male and female adults of varied ages and BMIs using multiple serum CIR_AA_s and participant characteristics. Multiple CIR_AA_s were highly correlated with animal protein intake and the APR, and this may have attenuated the responses of CIRs in NEAAs, especially the CIR_Ala_, to AS intake. The relationship between AS intake and the CIR_Ala_ was smaller in this study than in certain prior studies, although it was consistent with another controlled feeding study where the distribution of AS intake was restricted and SSB intake was low. Further research is needed in populations with higher and more diverse AS intake to determine whether CIR_AA_s can serve as biomarkers of AS intake in these populations. Finally, studies with larger sample sizes are needed to assess the stability of an AS predictive biomarker and its ability to correct for self-report measurement error.

## Figures and Tables

**Table 1:** Demographic, dietary, and amino acid carbon isotope ratio (CIR_AA_) characteristics of study participants (*n* = 99).

Characteristic	Values ^1^
Sex, female, *n*	54
Age, years	38.0 (29.5, 52.5)
Race/ethnicity ^2^, non-Hispanic White, *n*	77
BMI, kg/m^2^	26.9 (23.8, 30.2)
Body weight, kg	76.9 (67.7, 87.4)
Physical activity, active MET-h/d ^3^	12.4 (8.1, 15.8)
Nutrient intakes	
Energy, kcal/day	2516 (2279, 3020)
Carbohydrate, g/day	282.1 (238.8, 327.7)
Total sugar, g/day	110.0 (92.9, 135.7)
Added sugar, g/day	65.2 (44.7, 81.4)
Added sugar, % energy	9.5 (7.2, 12.4)
Added sugar to total sugars ratio (ASR)	0.59 (0.48, 0.68)
Protein, g/day	103.7 (91.3, 128.6)
Animal protein, g/day	66.9 (55.2, 87.9)
Animal protein to total protein ratio (APR)	0.66 (0.59, 0.70)
Food intakes	
Sugar-sweetened beverage (SSB), servings/day ^4^	0.42 (0.11, 0.97)
0 servings/day, *n*	15
<0.5 servings/day, *n*	42
≥0.5 servings/day, *n*	42
Corn, g/day ^5^	25.3 (13.5, 36.8)
Red meat, g/day ^6^	60.2 (27.3, 110.9)
Poultry, g/day ^6^	88.0 (44.6, 124.5)
Eggs, g/day	38.6 (21.9, 74.2)
Dairy, servings/day ^7^	1.6 (1.2, 2.3)
Fish/seafood, g/day	10.4 (0.0, 25.0)
0 g/day, *n*	37
<32 g/day, *n*	42
≥32 g/day, *n*	20
Amino acid CIR, δ^13^C, ‰ ^8^	
Alanine (Ala)	−20.1 (−21.0, −19.4)
Aspartic acid/asparagine (Asx)	−18.4 (−19.1, −17.4)
Glutamic acid/glutamine (Glx)	−16.5 (−17.2, −15.8)
Glycine (Gly)	−9.9 (−11.3, −7.7)
Histidine (His)	−13.7 (−14.7, −12.6)
Isoleucine (Ile)	−19.7 (−20.7, −18.6)
Leucine (Leu)	−27.4 (−27.9, −26.9)
Lysine (Lys)	−18.2 (−18.9, −17.4)
Methionine (Met)	−26.1 (−26.9, −25.3)
Phenylalanine (Phe)	−25.4 (−25.7, −24.9)
Proline (Pro)	−16.3 (−16.9, −15.6)
Serine (Ser)	−10.0 (−10.9, −8.9)
Threonine (Thr)	−3.4 (−4.3, −2.7)
Tyrosine (Tyr)	−24.8 (−25.4, −24.2)
Valine (Val)	−24.3 (−25.0, −23.7)

^1^ Values are *n*, where indicated, or median (25th, 75th percentile). ^2^ Others were Hispanic/Latino (*n* = 12), Asian (*n* = 4), African American (*n* = 3), Pacific Islander (*n* = 1), Native American (*n* = 1), and declined to answer (*n* = 1). ^3^ Active defined as metabolic equivalent of task (MET) > 2.5 and excludes sedentary and light-intensity physical activity. ^4^ One SSB serving = 8 fl oz (236.6 mL). ^5^ Excluding added sugars. ^6^ Including processed meat. ^7^ One dairy serving = 1 cup (236.6 mL) milk or 1.5 oz (42.5 g) natural cheese or 2 oz (56.7) processed cheese. ^8^ CIRs reported as per mil (‰) δ^13^C values, where δ^13^C = (R_sample_/R_standard_ − 1) × 1000‰; R = ^13^C/^12^C; the standard is Vienna Pee Dee Belemnite.

**Table 2:** Correlations of nonessential amino acid CIRs with 15-day mean dietary intakes (*n* = 99) ^1^.

	CIR_Ala_	CIR_Asx_	CIR_Glx_	CIR_Gly_	CIR_Pro_	CIR_Ser_
Nutrient intakes ^2^						
Carbohydrate, g/day	−0.05	−0.14	−0.17	−0.11	−0.38 *^4^	−0.09
Total sugar, g/day	0.10	−0.01	−0.05	−0.07	−0.15	−0.02
Total carbohydrate minus total sugar, g/day	−0.16	−0.21	−0.22	−0.11	−0.47 **	−0.12
Added sugar, g/day	0.32 *	0.22	0.15	0.07	0.04	0.16
Total sugar minus added sugar, g/day	−0.34 *	−0.39 *	−0.38 *	−0.27	−0.37 *	−0.31 *
ASR	0.40 **	0.33 *	0.30 *	0.19	0.23	0.25
Protein, g/day	−0.02	0.20	0.19	−0.01	−0.09	0.21
Animal protein, g/day	0.25	0.54 **	0.58 **	0.13	0.40 **	0.47 **
Plant protein, g/day	−0.46 **	−0.48 **	−0.52 **	−0.28	−0.61 **	−0.39 *
APR	0.48 **	0.70 **	0.75 **	0.30 *	0.61 **	0.62 **
Food intakes ^3^						
Corn, g/day	0.27	0.26	0.29	−0.01	0.08	0.16
Red meat, g/day ^4^	0.37 *	0.68 **	0.63 **	0.15	0.34 *	0.45 **
Poultry, g/day ^4^	0.12	0.29	0.32 *	0.10	0.21	0.32 *
Eggs, g/day	−0.02	0.22	0.25	0.00	0.04	0.20
Dairy, servings/day ^5^	0.20	0.18	0.25	0.11	0.11	0.28

^1^ The Bonferroni-adjusted significance level for these Pearson correlations is 0.0033 (=0.05/15), adjusting for the 15 dietary variables that were examined. * *p* < 0.0033; ** *p* < 0.0001. ^2^ The following variables were transformed as ln (g/day): protein, animal protein, and plant protein. ^3^ The following variables were Box–Cox-transformed: red meat, poultry, and dairy (λ = 0.67); corn and eggs (λ = 0.5). ^4^ Including processed meat. ^5^ One serving = 1 cup of milk, 1.5 oz. natural cheese, 2 oz. processed cheese, etc.

**Table 3:** Correlations of essential amino acid CIRs with 15-day mean dietary intakes (*n* = 99) ^1^.

	CIR_His_	CIR_Ile_	CIR_Leu_	CIR_Lys_	CIR_Met_	CIR_Phe_	CIR_Thr_	CIR_Tyr_	CIR_Val_
Nutrient intakes ^2^									
Carbohydrate, g/day	−0.11	−0.10	−0.31 *^4^	−0.16	−0.09	−0.30 *	−0.16	−0.21	−0.28
Total sugar, g/day	−0.13	−0.02	−0.18	−0.03	0.06	−0.14	−0.02	−0.12	−0.14
Total carbohydrate minus total sugar, g/day	−0.07	−0.15	−0.33 *	−0.23	−0.19	−0.35 *	−0.23	−0.22	−0.31 *
Added sugar, g/day	0.01	0.10	0.01	0.18	0.19	0.08	0.15	0.07	0.07
Total sugar minus added sugar, g/day	−0.29 *	−0.20	−0.40 **	−0.37 *	−0.20	−0.43 **	−0.29 *	−0.38 *	−0.42 **
ASR	0.17	0.20	0.21	0.37 *	0.25	0.29	0.29	0.26	0.29
Protein, g/day	0.24	−0.02	0.23	0.09	0.10	0.11	0.03	0.21	0.15
Animal protein, g/day	0.51 **	0.14	0.71 **	0.57 **	0.50 **	0.64 **	0.42 **	0.65 **	0.64 **
Plant protein, g/day	−0.24	−0.25	−0.54 **	−0.52 **	−0.44 **	−0.65 **	−0.49 **	−0.51 **	−0.58 **
APR	0.51 **	0.28 *	0.84 **	0.70 **	0.62 **	0.85 **	0.59 **	0.79 **	0.81 **
Food intakes ^3^									
Corn, g/day	0.19	−0.07	0.22	0.26	0.30*	0.25	0.13	0.19	0.17
Red meat, g/day ^4^	0.51 **	0.18	0.65 **	0.63 **	0.52 **	0.60 **	0.41 **	0.64 **	0.58 **
Poultry, g/day ^4^	0.26	0.19	0.43 **	0.18	0.15	0.36 *	0.20	0.36 *	0.38 *
Eggs, g/day	0.31 *	−0.06	0.27	0.08	0.00	0.20	0.01	0.27	0.21
Dairy, servings/day ^5^	0.02	−0.06	0.12	0.28 *	0.38 *	0.22	0.26	0.17	0.19

^1^ The Bonferroni-adjusted significance level for these Pearson correlations is 0.0033 (=0.05/15), adjusting for the 15 dietary variables that were examined. * *p* < 0.0033; ** *p* < 0.0001. ^2^ The following variables were transformed as ln (g/day): protein, animal protein, and plant protein. ^3^ The following variables were Box–Cox-transformed: red meat, poultry, and dairy (λ = 0.67); corn and eggs (λ = 0.5). ^4^ Including processed meat. ^5^ One serving = 1 cup of milk, 1.5 oz. natural cheese, 2 oz. processed cheese, etc.

**Table 4:** Model of added sugar intake based on participant characteristics and serum CIR_AA_s in a controlled feeding study of men and women (*n* = 99).

Model Term	β	SE	*p*-Value	*R* ^2^	Adjusted *R*^2^
Intercept	60.43	83.48	0.471	0.38	0.32
Body weight	0.47	0.24	0.054		
Sex	−11.77	6.49	0.073		
CIR_Ala_	22.94	3.94	<0.001		
CIR_Val_	−18.03	5.16	0.001		
CIR_Lys_	12.55	4.44	0.006		
CIR_Glx_	−15.88	5.99	0.009		
CIR_Ser_	6.89	2.77	0.015		
CIR_Gly_	−5.00	2.04	0.016		

## Data Availability

Data described in the manuscript, code book, and analytical code will be made available upon request pending application and approval.

## Supplementary Materials

The following supporting information can be downloaded at: https://www.mdpi.com/article/10.3390/nu14204308/s1, Figure S1: Study participant flow chart; Table S1: Amino acids used in the external standard and purchased from Sigma-Aldrich (St. Louis, MO, USA); Table S2: Analytical error and between-batch reproducibility of CIR_AA_ measurements in the check (QA) sample.; Table S3: Correlation matrix of CIR_AA_s (*n* = 99); Table S4: Frequency of covariate selection in 2000 bootstrap samples.

## Appendix A. Alternative Model Selection Approaches and Comparison

We selected added sugar (AS) intake models using additional approaches: regression shrinkage and selection using LASSO [27] and random forest regression [28]. The covariates considered were three participant characteristics (sex, age, and body weight) and 15 amino acid carbon isotope ratios (CIR_AA_s). We calculated the average mean square prediction error (MSPE) over ten runs of each method and compared these MSPEs across methods. For each run, the full data set (*n* = 99) was stratified and randomly split into training (*n* = 60) and testing (*n* = 39) subsets, ensuring an even distribution of the covariates between subsets. The same ten data splits were used across the three methods.

For both forward selection and LASSO, cross-validation within the training subset was used to select hyperparameters: the number of covariates chosen for forward selection and the regularization parameter for LASSO. For the forward selection method, we used 10-fold cross-validation to select the number of steps. For LASSO, we used 5-fold cross-validation to choose the regularization parameter, which restricts the sum of absolute values of the regression coefficients. After hyperparameters were selected, model selection with those parameters (via forward selection or LASSO) was performed on the full training subset. Selected models were applied to the testing subset in order to calculate the MSPE.

Within each run of the random forest approach, 500 decision trees (the “forest”) were built using the training subset. These trees were applied to the testing subset, and the output was averaged to calculate the MSPE.

**Table A1:** MSPEs of three selection approaches to modeling AS intake ^1^.

Run Number	AS Variance	Forward Selection	LASSO	Random Forest
1	1348.08	1119.92	1107.32	1196.25
2	1355.08	992.72	1242.55	1241.18
3	675.59	996.41	808.36	819.78
4	914.26	1387.35	914.88	802.74
5	817.77	695.8	798.21	756.23
6	1015.65	1499.37	1141.52	1221.88
7	1453.29	1344.77	1331.30	1365.71
8	1333.65	1093.81	1246.11	1186.58
9	1797.72	1575.05	1676.61	1602.48
10	1366.57	1433.14	1417.67	1539.98
Average	1207.766	1213.834	1168.454	1173.282

^1^ AS intake (g/day) and covariates were not transformed. AS, added sugars; MSPE, mean square prediction error.

There was low model stability across selection approaches, as indicated by variable MSPEs across runs (Table A1) and variable numbers of covariates in the forward selection and LASSO runs. The ratio of MSPE to AS variance also varied across runs, and MSPE was often higher or nearly equal to AS variance, indicating very low predictive ability of selected models. Average MSPE did not vary greatly across the three selection approaches.
