# Supplementary material for: Evaluating a Model of Added Sugar Intake Based on Amino Acid Carbon Isotope Ratios in a Controlled Feeding Study of U.S. Adults
Source: Nutrients. 2022 Oct 14;14(20):4308. doi: 10.3390/nu14204308 (PMC9611411; doi:10.3390/nu14204308)
Supplement: Supplementary file 1 [file nutrients-14-04308-s001.zip › nutrients-1930655-supplementary.pdf]

Evaluating a model of added sugar intake based on amino acid carbon isotope ratios in a controlled feeding study of U.S. adults. J. J. Johnson. Supplementary Material.

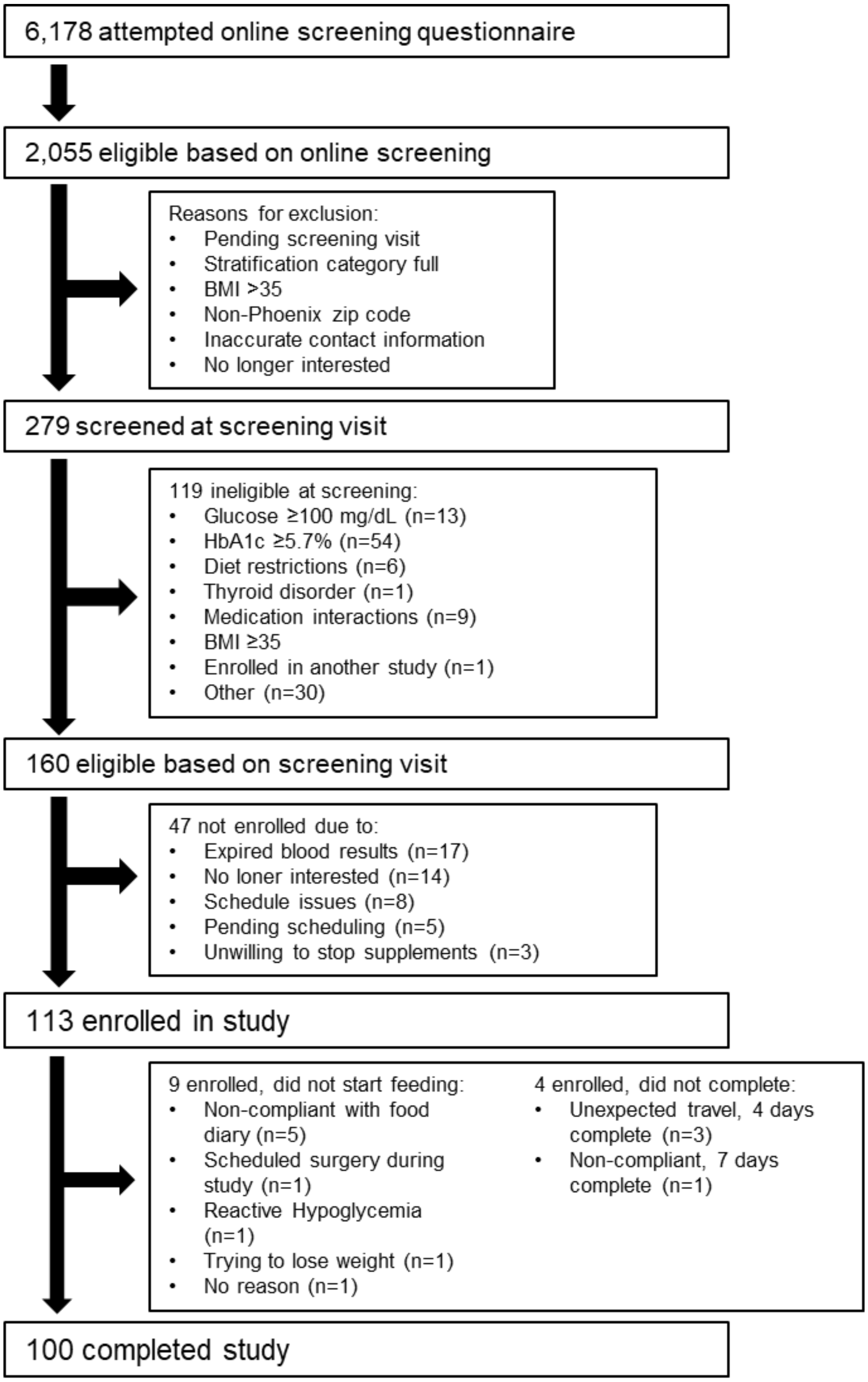

Figure S1: Study participant flow chart. Adapted with permission from [1].

Table S1: Amino acids used in the external standard and purchased from Sigma-Aldrich (St. Louis, MO, USA).

Amino acid	Lot number	CIR[1] (‰)
L-Alanine	BCBN6412V	-19.17
L-Aspartic acid	BCBN3442V	-27.29
L-Glutamine	BCBM0517V	-28.89
Glycine	SLBS1578V	-42.27
L-Histidine	BCBL8528V	-9.44
L-Isoleucine	BCBD5312V	-11.24
L-Leucine	BCBM2322V	-28.93
L-Lysine	BCBR4746V	-28.13
L-Methionine	BCBQ3447V	-33.93
L-Phenylalanine	SLBQ7928V	-12.18
L-Proline	BCBP4505V	-12.24
L-Serine	1349921V	-13.60
L-Threonine	BCBM6171V	-10.41
L-Tyrosine	BCBP3212V	-24.12
L-Valine	BCBQ2367V	-10.75

[1] Measured at the Alaska Stable Isotope Facility using elemental analyzer isotope ratio mass spectrometry. CIR (carbon isotope ratio), reported as the $\delta^{13}C$ value in units of ‰ where $\delta^{13}C = (R_{sample}/R_{standard} - 1) \times 1000)$, $R = {}^{13}C/{}^{12}C$, and the standard is VPBD).

Table S2: Analytical error and between-batch reproducibility of CIR_{AA} measurements in the check (QA) sample[1].

CIR	Within batch SEM (‰)[2]	Between batch SD (‰)[3]
Ala	0.13	0.49
Gly	0.25	1.33
Val	0.07	0.28
Leu	0.10	0.29
Ile	0.14	1.12
Thr	0.20	1.08
Ser	0.26	1.07
Asx	0.18	0.63
Pro	0.19	0.78
Glx	0.04	0.34
Met	0.22	0.80
Phe	0.05	0.18
Lys	0.31	0.51
Tyr	0.12	0.53
His	0.33	1.09

[1] CIR_{AA}, carbon isotope ratio of amino acids; QA, quality assurance

[2] Mean of propagated SEMs of multiple injections of AA standard and the QA sample across analytical batches (n = 8), calculated as follows:

$$SEM\ CIR_{AA,smp} = \frac{1}{p} \times \left(\frac{SD_{d,smp}^2}{n_{d,smp}} + \frac{SD_{d,std}^2}{n_{d,std}}\right)^{1/2}$$

where, p is the proportion of carbon in the derivatized AA from the un-derivatized AA; $SD_{d,smp}$ is the standard deviation of the measured CIR_{AA} in the replicate injections of the sample; $n_{d,smp}$ is the number of injections per sample; $SD_{d,std}$ is the standard deviation of the measured CIR_{AA} in the external standard; and, $n_{d,std}$ is the number of injections of the external standard [2]

[3] SD of QA sample across analytical batches (n = 8)

Table S3: Correlation matrix of CIR$_{AAs}$ (n = 99)[1].

	Ala	Gly	Val	Leu	Ile	Thr	Ser	Asx	Pro	Glx	Met	Phe	Lys	Tyr	His
**Ala**	1	0.71	0.73	0.49	0.51	0.53	0.48	0.52	0.64	0.63	0.53	0.60	0.51	0.36	0.24
**Gly**	---	1	0.57	0.25	0.66	0.54	0.50	0.29	0.58	0.32	0.18	0.40	0.25	0.06	0.01
**Val**	---	---	1	0.87	0.52	0.72	0.65	0.71	0.80	0.78	0.67	0.91	0.76	0.74	0.51
**Leu**	---	---	---	1	0.25	0.56	0.60	0.82	0.66	0.81	0.65	0.91	0.82	0.87	0.66
**Ile**	---	---	---	---	1	0.49	0.29	0.13	0.55	0.14	0.13	0.33	0.16	0.11	-0.01
**Thr**	---	---	---	---	---	1	0.72	0.56	0.73	0.57	0.59	0.75	0.56	0.49	0.17
**Ser**	---	---	---	---	---	---	1	0.73	0.62	0.66	0.50	0.71	0.53	0.52	0.25
**Asp**	---	---	---	---	---	---	---	1	0.57	0.88	0.58	0.80	0.79	0.76	0.58
**Pro**	---	---	---	---	---	---	---	---	1	0.6	0.58	0.79	0.56	0.50	0.24
**Glu**	---	---	---	---	---	---	---	---	---	1	0.65	0.84	0.82	0.77	0.62
**Met**	---	---	---	---	---	---	---	---	---	---	1	0.72	0.63	0.65	0.33
**Phe**	---	---	---	---	---	---	---	---	---	---	---	1	0.83	0.84	0.53
**Lys**	---	---	---	---	---	---	---	---	---	---	---	---	1	0.82	0.66
**Tyr**	---	---	---	---	---	---	---	---	---	---	---	---	---	1	0.67
**His**	---	---	---	---	---	---	---	---	---	---	---	---	---	---	1

[1] Pearson correlation coefficients are shown. CIR$_{AA}$, carbon isotope ratio of amino acid.

Table S4: Frequency of covariate selection in 2,000 bootstrap samples[1].

Covariate	Selected (%)
Body weight*[2]	67.4
Sex*	54.0
Age	13.4
Ala*	97.7
Gly*	73.2
Val*	67.6
Leu	57.5
Ile	38.2
Thr	27.6
Ser*	45.6
Asx	42.2
Pro	25.2
Glx*	59.4
Met	26.7
Phe	22.2
Lys*	76.0
Tyr	33.4
His	28.2

[1] Bootstrap of two-step forward selection based on AIC.

[2] Asterisked covariates were selected in the final AS model.

References

1. Tasevska, N.; Sagi-Kiss, V.; Palma-Duran, S.A.; Barrett, B.; Chaloux, M.; Commins, J.; O'Brien, D.M.; Johnston, C.S.; Midthune, D.; Kipinis, V.; et al. Investigating the performance of 24-h urinary sucrose and fructose as a biomarker of total sugars intake in US participants–a controlled feeding study. *Am. J. Clin. Nutr.* **2021**, *114*, 721–730. https://doi.org/10.1093/ajcn/nqab158.
2. O'Brien, D.M.; Boggs, C.L.; Fogel, M.L. The amino acids used in reproduction by butterflies: A comparative study of dietary sources using compound-specific stable isotope analysis. *Physiol. Biochem. Zool.* **2005**, *78*, 819–827. https://doi.org/10.1086/431191.
